# Gain Analysis of Self-Fitting Over-the-Counter Hearing Aids: A Comparative and Longitudinal Analysis

**DOI:** 10.3390/audiolres15010017

**Published:** 2025-02-13

**Authors:** Megan Knoetze, Vinaya Manchaiah, Kayla Cormier, Carly Schimmel, Anu Sharma, De Wet Swanepoel

**Affiliations:** 1 Department of Speech-Language Pathology and Audiology, University of Pretoria, Lynnwood Road & Roper Street, Pretoria 0028, South Africa; megan.knoetze@up.ac.za (M.K.); vinaya.manchaiah@cuanschutz.edu (V.M.); 2 Virtual Hearing Lab, a Collaborative Initiative Between the University of Colorado School of Medicine, Aurora, CO 80045, USA, and the University of Pretoria, Pretoria, South Africa; 3 Department of Otolaryngology-Head and Neck Surgery, University of Colorado School of Medicine, Aurora, CO 80045, USA; 4 UCHealth Hearing and Balance, University of Colorado Hospital, Aurora, CO 80045, USA; 5 Department of Speech and Hearing, School of Allied Health Sciences, Manipal Academy of Higher Education, Manipal 576104, India; 6 Department of Speech, Language, and Hearing Sciences, University of Colorado Boulder, Boulder, CO 80309, USA; kayla.cormier@colorado.edu (K.C.); carly.schimmel@colorado.edu (C.S.); anu.sharma@colorado.edu (A.S.)

**Keywords:** over the counter, hearing aids, gain, real-ear measurements

## Abstract

Objectives: To investigate the gain provided by self-fitting over-the-counter (SF-OTC) hearing aids compared to clinical NAL-NL2 targets, the differences between various FDA-approved SF-OTC devices, and potential changes in gain over time. Methods: Two experiments were conducted: (1) a cross-sectional comparison of six SF-OTC hearing aids (n = 43) and (2) a longitudinal evaluation of gain within five days of self-fitting and four additional time points (n = 15). Real-ear measurements (REMs) were used to measure gain. Results: SF-OTC hearing aid gain corresponded with 10 dB SPL but not 5 dB SPL criteria from NAL-NL2 targets. Differences between NAL-NL2 targets and gain did not differ significantly between devices. There were no significant changes in gain over time for any input level. Conclusions: SF-OTC hearing aids generally provide user-selected gain levels lower than clinical targets, particularly at higher frequencies. The gain remains stable over time, indicating limited user adjustment after initial fitting. OTC hearing aid manufacturers should consider implementing fitting algorithms that allow for gradual user acclimatization.

## 1. Introduction

Over-the-counter (OTC) hearing aids have emerged as a promising solution to the growing demand for accessible and affordable hearing care [1]. These devices, purchased without a prescription, are designed to improve the hearing of adults with self-perceived mild-to-moderate hearing loss without extensive clinical intervention, thus increasing the public’s access to hearing aids [1]. Initial research on OTC hearing aids has demonstrated that they provide equivalent benefits and satisfaction to audiologist-fit hearing aids in both short-term and long-term outcomes [2,3]. Therefore, OTC hearing aids offer a cost-effective alternative to traditional, prescription-based devices.

Prescription hearing aids for adults are typically fitted by hearing healthcare professionals using either the manufacturer’s fitting rationale or the National Acoustic Laboratories—Non-Linear 2 (NAL-NL2) prescription formula, which is designed to maximize speech intelligibility and listening comfort [4]. To ensure these hearing aids provide the appropriate level of gain for an individual’s hearing loss, real-ear measurements (REMs) are widely regarded as the best clinical practice [5]. REMs involve placing a probe microphone in the ear canal to measure the sound delivered by the hearing aid, thereby ensuring that it meets the prescribed amplification targets [5]. Studies highlight the importance of using REMs for fitting hearing aids, as they may lead to improved speech intelligibility, better self-perceived hearing aid benefit, higher sound quality ratings, and user preference [6,7,8,9,10]. While REMs statistically improve hearing aid outcomes, this conclusion is based on limited studies and participants, according to a recent systematic review [11]. The practical importance of these benefits remains to be determined, as minimum clinically significant differences have yet to be established [11].

In contrast, OTC hearing aids are typically self-fitted by users and the hearing aid gain is not verified by an audiologist conducting REMs [1]. Several clinical trials on self-fitting OTC (SF-OTC) hearing aids indicated that the gains measured through REMs were slightly below the NAL-NL2 targets, especially in the higher frequencies [3,12,13]. To our knowledge, no study has examined the gain provided by existing OTC hearing aids on the market or tracked these measurements longitudinally to assess any potential changes resulting from user adjustments or acclimatization. Moreover, Shah et al. [14] highlighted significant challenges in the readability and accessibility of online information related to OTC hearing aids.

In a study by Keidser et al. [15], new users of prescription hearing aids were given hearing programs with gain reductions from NAL-NL1 targets and instructed to use the volume control in addition to these programs. Over a period of 13 months, these users exhibited gain acclimatization, characterized by a decrease in volume control usage. In contrast, other studies have noted that when participants can request changes to their prescription hearing aid gain, only 15–20% of participants provide feedback, resulting in gain changes [10,16]. Therefore, exploration of OTC hearing aid gain over time is expected. Furthermore, the FDA OTC rule states that the user must be able to adjust the hearing aids by themself. As a result, most OTC hearing aids do not allow clinicians to adjust gains based on hearing sensitivity (i.e., the pure-tone audiogram). It is essential to understand what gain they provide and how it relates to the clinical standard based on NAL-NL2. Therefore, this study aims to address this gap by investigating (1) how the gain in SF-OTC devices compares with clinical NAL-NL2 targets, (2) whether it differs between various FDA-approved SF-OTC devices, and (3) if the gain in SF-OTC devices changes over time.

## 2. Method

### 2.1. Study Design

Ethical clearance was obtained from the University of Pretoria Humanities Research Ethics Committee (HUM021/1122). This study included two experiments from larger clinical trials. The first experiment compared the usability and performance of six SF-OTC hearing aids—HP Hearing PRO, Jabra Enhance Plus, Lexie B2 Powered by Bose, Lexie Lumen, Sontro, and Sony CRE-C10—using a cross-sectional design and REMs to measure gain after self-fitting. The second experiment used a longitudinal design with three SF-OTC hearing aids: Lexie B2, Lexie Lumen, and Soundwave Sontro. REMs measured gain within five days of self-fitting and at four additional points over the first year of use. All participants provided written informed consent.

### 2.2. Participants

#### 2.2.1. Experiment 1—Comparison of REMs Across SF-OTC Devices

A total of 43 adults who reported having mild-to-moderate hearing difficulties and no active outer and middle ear pathologies were recruited using purposive sampling (Knoetze et al., In Press) [17]. A high level of English proficiency was determined through an online test [18]. Employing a Latin square method, 29 participants were assigned to two groups for self-fitting devices to mitigate order effects. An additional 14 participants were recruited to self-fit the HP Hearing PRO and Lexie Lumen or Sony CRE-C10 devices, resulting in 13 to 15 users per device, with each participant fitting two devices. Of the 43 participants, 5 reported previous hearing aid use. The study included 24 male and 19 female participants, with a mean age of 59.7 years (SD 14.3). The mean Pure-Tone Average (PTA) for frequencies 500, 1000, 2000 and 4000 Hz was 36.5 dB HL (SD 16.5) for the left ear and 32.8 dB HL (SD 16.7) for the right ear (see Supplementary Figure S1).

#### 2.2.2. Experiment 2—Longitudinal Evaluation of REMS in SF-OTC Hearing Aids

Purposive sampling resulted in fifteen adults participating in this study with bilateral mild to moderate high frequency sensorineural hearing loss. All participants had a hearing loss of more than 25 dB, starting at a minimum of 2000 Hz. Individuals with conductive and mixed hearing loss were not included in the study. The mean PTA for frequencies 500, 1000, 2000, and 4000 Hz was 37.8 dB HL (SD 14.8) for the left ear and 36.5 dB HL (SD 15.8) for the right ear (Supplementary Figure S2). Five participants used Soundwave Sontro, six used Lexie B2, and four used Lexie Lumen. All participants were new hearing aid users (no prior experience). Six participants were male; the remaining nine were female. The average age was 70.5 years (SD 7.0).

### 2.3. Data Collection

#### 2.3.1. Experiment 1—Comparison of REMs Across SF-OTC Devices

Experiment 1 was conducted in South Africa. Participants underwent hearing assessments by a certified audiologist before self-fitting their hearing aids, including otoscopy, tympanometry, audiometry, and speech-in-noise tests (Digits-in-Noise, QuickSIN). Using an iPhone X provided by the researcher and manufacturer instructions, participants self-fitted their hearing aids. If participants encountered difficulties during the self-fitting process, they could seek assistance from either the researcher or a family member who accompanied them. REMs were conducted after each fitting for average, soft, and loud speech (65, 55, and 75 dB SPL) using the International Speech Test Signal (ISTS). Most measurements were taken with Audioscan Verifit1; two participants’ REMs were performed with the MedRx system due to equipment availability.

#### 2.3.2. Experiment 2—Longitudinal Evaluation of REMS in SF-OTC Hearing Aids

Experiment 2 took place in Colorado, United States of America. Each participant underwent a hearing test before obtaining the SF-OTC hearing aids. Two licensed audiologists conducted the tests in a sound-proof booth, including otoscopic examination, pure-tone audiometry, speech reception thresholds, word recognition scores, and unaided speech-in-noise tests (DIN and QuickSIN). The results confirmed mild to moderate sensorineural hearing loss and were used to generate real-ear fitting targets. Participants self-fitted their hearing aids using a smartphone app on their own device, with no additional support beyond what the manufacturers provided. They were instructed to use the app to self-fit the hearing aids and make adjustments, such as volume or sound preferences, as needed. No further guidance was given regarding adjustments over time to minimize external influence. While participants were able to modify settings, such as volume, through the app, the study did not track or record the specific adjustments made throughout the study period. To measure the gain, REMs were measured within five days of self-fitting the hearing aids and at four subsequent intervals over the first year of hearing aid use. The Audioscan Verifit 1 was used to measure the gain through REMs for average, soft, and loud speech (65, 55, and 75 dB SPL) using the ISTS. 

Follow-up testing occurred at approximately 1.25 months (SD 0.45 months), 3.73 months (SD 0.47 months), 5.73 months (SD 0.47 months), and 12.8 months (SD 0.92) of hearing aid use. Two participants withdrew before 1 month, and another withdrew before 3 months due to lack of perceived benefit. One participant missed the 3–4-month follow-up, and another missed the 5–6-month visit. Two did not complete the one-year follow-up. However, available data were included in the analyses.

### 2.4. Data Analysis

Data analysis was conducted using IBM SPSS 28.0.1.0. Descriptive statistics (e.g., mean and standard deviation) were calculated for both experiments. The Shapiro–Wilk test assessed normality, guiding the choice between non-parametric and parametric tests. In this study, the NAL-NL2 targets were generated using default parameters for adults with hearing loss. The targets served as a reference for comparison with user-selected gain measured through REMs and were not customized to individual participant characteristics, such as experience level or device-specific parameters. For Experiment 1, we first calculated the difference between NAL-NL2 targets and REM-measured gain for each frequency in both ears across three input levels. The analysis focused on the gain difference at each frequency within the spectrum. Due to non-normal distribution (*p* < 0.05), the Kruskal–Wallis *H* test and Dunn’s procedure with Bonferroni correction were used to compare differences between hearing aids.

For Experiment 2, we evaluated these gain differences at multiple time points (baseline, 1–2 months, 3–4 months, 5–6 months, and 12–15 months) for three input levels. Repeated measures ANOVA was applied where data were normally distributed (*p* > 0.05), with Greenhouse–Geisser correction used if sphericity was violated. For loud speech, where data were non-normal (*p* < 0.05), the Friedman test was used, with Bonferroni-adjusted post hoc comparisons.

Thereafter, Root Mean Square Error (RMSE) was calculated to quantify the fit of measured gain to NAL-NL2 targets in dB SPL, providing a single metric for error across frequencies (500–6000 Hz). Lower RMSE values indicate better alignment with clinical targets. RMSE values across 500–6000 Hz were calculated for all hearing aids at each input level and time point. RMSE values were derived by averaging the squared differences between the NAL-NL2 target and gain measured through REMs for frequencies 500, 1000, 2000, 3000, 4000, and 6000 Hz and then taking the square root of this average. In Experiment 1, the Kruskal–Wallis *H* test and Dunn’s procedure with Bonferroni correction were used for RMSE comparisons. In Experiment 2, repeated measures ANOVA was used for normally distributed data (*p* > 0.05), with Greenhouse–Geisser correction if needed, and the Friedman test for non-normal data (*p* < 0.05).

## 3. Results

### 3.1. Experiment 1—Comparison of REMs Across SF-OTC Devices

Table 1 compares the average differences between NAL-NL2 targets and gains measured through REMs for average speech, soft speech, and loud speech across different SF-OTC hearing aids. Although they do not correspond with the clinically accepted criteria of 5 dB, most hearing aids were within 10 dB of the NAL-NL2 targets for average and soft speech, considering both ears. However, Sontro was 10.4 (6.3) dB and 11.7 (7.0) dB below target on average. For loud speech, all hearing aids were within 10 dB of NAL-NL2 targets, considering both ears. For average speech, the correspondence to the 5 dB criteria across the hearing aids varied across frequencies. The frequency and percentage of measurements within 5 and 10 dB from NAL-NL2 targets for average speech are included in Supplementary Tables S1 and S2. Across all devices, low frequencies, such as 250 Hz (75.9%) and 500 Hz (52.4%), showed higher correspondence, while high frequencies, such as 4000 Hz (17.1%) and 6000 Hz (24.1%), exhibited lower correspondence to the 5 dB criteria.

**Figure 1:**
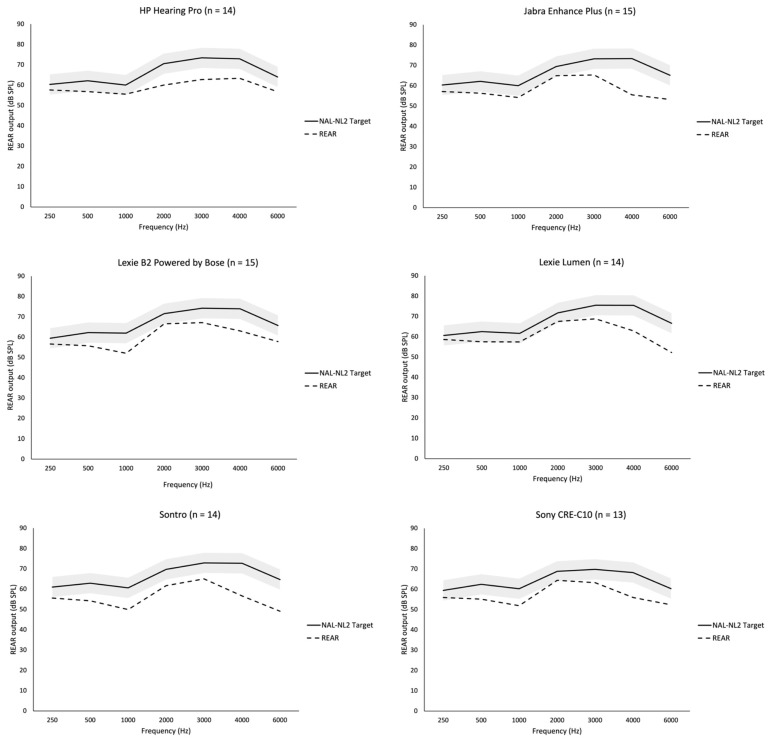
Comparison of average prescribed NAL-NL2 targets and average Real-Ear Aided Response (REAR) measured at 65 dB (average speech) for ears combined across OTC hearing aids. Shading indicates the tolerance limits (±5 dB) from the mean for NAL-NL2 prescriptive targets.

The differences between NAL-NL2 targets and gains measured through REMs did not vary significantly between the different hearing aids for average speech (*H*(5) = 6.840, *p* = 0.233), soft speech (*H*(5) = 7.335, *p* = 0.197), or loud speech (*H*(5) = 4.022, *p* = 0.546). Additionally, no specific pairwise differences were found using Dunn’s procedure with a Bonferroni correction for multiple comparisons. See Figure 1 and Supplementary Figures S3 and S4.

**Figure 2:**
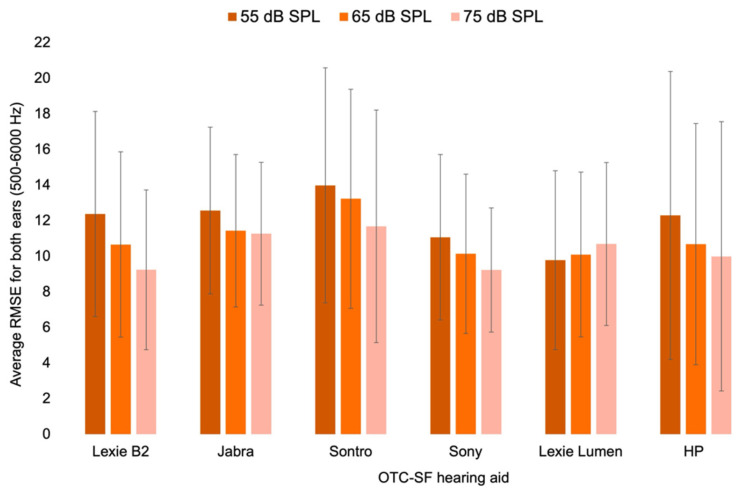
Average RMSE values for both ears (500–6000 Hz) in dB SPL across the various SF-OTC hearing aids and input levels. Note: Lower RMSE values indicate closer alignment to the NAL-NL2 target.

All of our measurements exceeded the acceptable RMSE threshold of 5 dB, which measures the goodness of fit to targets across different input levels and SF-OTC hearing aids [19]. The average RMSE for 500–6000 Hz for all SF-OTC hearing aids is presented in Supplementary Table S3, and Figure 2 illustrates average RMSE values (500–6000 Hz) across the various SF-OTC hearing aids and input levels. RMSE values, considering both ears, were not significantly different between the various hearing aids for average speech (*H*(5) = 7.077, *p* = 0.215), soft speech (*H*(5) = 8.314, *p* = 0.140), or loud speech (*H*(5) = 9.219, *p* = 0.101). Additionally, specific pairwise comparisons using Dunn’s procedure with a Bonferroni correction for multiple comparisons did not reveal any significant differences.

### 3.2. Experiment 2—Longitudinal Evaluation of REMS in SF-OTC Hearing Aids

The longitudinal evaluation of average differences between NAL-NL2 targets and gains measured through REMs after initial fit and at follow-up intervals is presented in Table 2. For average speech, no significant changes were observed in the average difference between NAL-NL2 targets and gains measured through REMs over time: *F*(2.358, 35.375) = 0.875, *p* = 0.441. Similarly, for soft speech, no significant changes were observed in the average difference between NAL-NL2 targets and gains measured through REMs over time: *F*(2.337, 35.048) = 1.376, *p* = 0.267. For loud speech, there were also no significant changes in the average difference between NAL-NL2 targets and gains measured through REMs over time (χ^2^ (4) = 3.022, *p* = 0.554).

**Figure 3:**
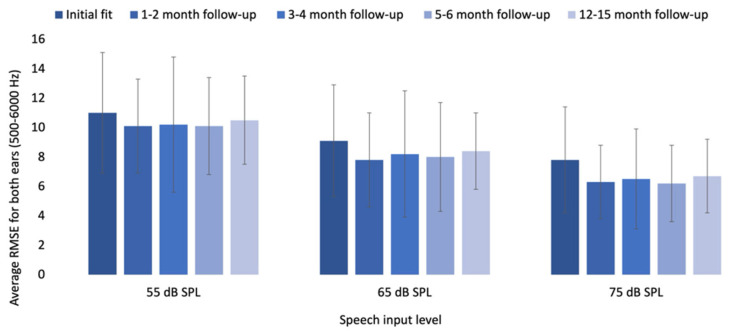
Average RMSE values for both ears (500–6000 Hz) in dB SPL across time points and input levels. Note: Lower RMSE values indicate closer alignment to the NAL-NL2 target.

All RMSE values exceeded the 5 dB criterion [19], consistent across different input levels and time points. Supplementary Table S4 presents the average RMSE for 500–6000 Hz after initial fit and at follow-up intervals, and Figure 3 illustrates average RMSE values (500–6000 Hz) across time points and input levels. For average speech, considering both ears, there were no significant changes in RMSE values over time: *F*(2.349, 35.235) = 1.448, *p* = 0.248. Similarly, in soft speech, considering both ears, there were no significant changes in RMSE values over time: *F*(4, 60) = 2.262, *p* = 0.073. For loud speech, the Friedman test also showed no significant changes in RMSE values over time: χ^2^ (4) = 4.750, *p* = 0.314.

## 4. Discussion

The present study highlights several important considerations regarding the self-fit gain of FDA-approved SF-OTC hearing aids compared to clinical targets, across devices, and in terms of changes over time. One key observation is that SF-OTC hearing aids generally provide user-selected gain lower than clinical NAL-NL2 targets, as previously reported [3,12,13]. Users self-fit their SF-OTC devices and might benefit from an adjustment phase despite the less precise initial gain settings. This lower gain may resemble the “comfort fit” provided during the acclimatization period for prescription hearing aids, where users gradually adapt to amplified sound. Keidser et al. [4] emphasized the importance of managing gain adaptation, highlighting that new hearing aid users could benefit from gradual adjustments to gain levels. While acclimatization has been widely investigated in prescription hearing aids, the self-fitting nature of OTC devices presents unique challenges. For self-fitting devices, acclimatization could involve algorithms that gradually increase gain levels over time based on user feedback, even in devices that do not include in situ hearing tests. Future studies could investigate the types of user feedback, such as subjective comfort ratings or perceived audibility in various listening environments, that may inform algorithms designed to gradually increase gain over time.

Additionally, the results showed that SF-OTC hearing aid gain, measured using REMs, was not significantly different between different device brands.

The longitudinal analysis showed that SF-OTC hearing aid gain does not change significantly over time, even when users have the ability to repeatedly perform in situ testing. This consistency in gain levels over time indicates that users did not make significant further adjustments after initially fitting their hearing aids. Furthermore, Almufarrij et al. [11] examined the preferences of new prescription hearing aid users who were initially fitted with significantly less gain and then adjusted using REMs to match the NAL-NL2 prescription targets. The study found that only 20% of participants requested gain adjustments to their hearing aids. Additionally, the reduced gain was rated as more comfortable, and hearing aid preferences remained stable over six weeks of use. Keidser et al. [15] also demonstrated that new hearing aid users prefer progressively less overall gain for average input levels than experienced HA users with a similar degree of hearing loss as the hearing loss increases. Their findings also showed that new users may gradually become accustomed to their hearing aids’ settings over time, thereby requiring fewer adjustments, such as using the volume control, as they adapt to the device. These findings suggest that future studies should explore hearing aid gain acclimatization and consider fitting formulas that automatically increase SF-OTC gain over time.

Most OTC hearing aid manufacturers generally do not provide software for audiologists to program these devices, except for manufacturers such as Sennheiser (All Day Clear), Eargo, and Jabra. This lack of professional programmability limits the ability to achieve a fit to the clinical NAL-NL2 targets. While some adjustments to gain can be made on specific OTC devices by performing REMs and using an app to tweak the settings, these adjustments are limited. A recent study by Manchaiah et al. [20] indicates that app-based adjustments may not fully replicate the precision achieved through professional fitting and REMs. While the effect of reduced precision on outcomes is still unclear, this may suggest room for hybrid models where a healthcare professional could support OTC users who report a need for more precise adjustments. The option of such a model could supplement user control with some professional assistance to support improved outcomes.

There is also increasing interest in comparing OTC and clinically fitted hearing aids through technical measurements. Initiatives such as HearAdvisor and other independent laboratories are evaluating how well these devices meet prescribed targets, providing valuable insights into their performance and potential areas for improvement. Despite the lower gain provided by SF-OTC hearing aids compared to NAL-NL2 targets, the studies published to date report good outcomes with these devices, including self-reported and speech-in-noise benefits [3,12,13,21]. This suggests that for adults, having slightly lower gain than prescription targets may result in similar outcomes, raising questions about what constitutes “optimal” targets. User comfort and subjective experience appear to play a significant role in the overall effectiveness of hearing aids. In line with Almufarrij et al. [11], this highlights the need for more research to better inform and potentially redefine optimal targets for hearing aid performance. These findings prompt a re-evaluation of existing standards such as NAL-NL2, suggesting that updates or new approaches could be beneficial in ensuring the best outcomes for users.

This study has a few limitations. First, the missing data in Experiment 2 may affect the interpretation of the results. Despite efforts to minimize data loss, the absence of complete data for all participants should be considered when drawing conclusions. Additionally, this study did not track the specific adjustments participants made to their hearing aids, such as changes to volume or sound settings through the app. This lack of data limits our ability to fully understand how user interactions may have influenced the results. Furthermore, while Experiment 1 was conducted in South Africa, Experiment 2 took place in the USA. These geographic differences may impact the generalizability of the findings across different regions.

## 5. Conclusions

This study shows that SF-OTC hearing aid gain is typically lower than NAL-NL2 targets; SF-OTC hearing aid gain does not differ significantly between different FDA-approved SF-OTC devices and remains stable over time. Despite the lower gain compared to NAL-NL2 targets, positive user outcomes in recent clinical trials on SF-OTC hearing aids highlight the importance of subjective experience and comfort. The study results provide additional evidence that may contribute to the ongoing discussion of what constitutes the optimal gain for hearing aid fitting. While the findings are based on technical measures like REMs, they highlight the need to further explore the balance between prescribed gain targets and user satisfaction to optimize hearing aid technology. Additionally, further research is needed to determine the optimal gain for adults with hearing loss.

## Figures and Tables

**Table 1:** Experiment 1: Average differences between NAL-NL2 targets and real-ear measurements in dB SPL across six self-fitting over-the-counter hearing aids.

	Average Speech at 65 dB SPL Mean (SD)			Soft Speech at 55 dB SPL Mean (SD)			Loud Speech at 75 dB SPL Mean (SD)		
Hearing Aid	Left Ear	Right Ear	Both Ears	Left Ear	Right Ear	Both Ears	Left Ear	Right Ear	Both Ears
HP Hearing Pro (n = 14)	3.9 (6.3)	10.5 (8.0)	7.2 (7.8)	6.0 (7.5)	12.5 (9.0)	9.3 (8.8)	4.0 (8.3)	8.5 (7.3)	6.3 (8.0)
Jabra Enhance Plus (n = 15)	8.4 (5.7)	7.9 (4.2)	8.1 (4.9)	9.7 (6.3)	9.9 (4.0)	9.8 (5.2)	6.8 (5.4)	6.5 (3.8)	6.6 (4.6)
Lexie B2 (n = 15)	7.3 (7.4)	7.0 (6.1)	7.2 (6.7)	10.0 (8.2)	8.7 (6.4)	9.3 (7.3)	5.5 (5.9)	5.4 (4.9)	5.5 (5.3)
Lexie Lumen (n = 14)	8.0 (5.2)	6.0 (4.1)	7.0 (4.7)	8.4 (5.6)	6.2 (5.0)	7.3 (5.3)	7.5 (5.1)	5.7 (3.6)	6.6 (4.4)
Soundwave Sontro (n = 14)	12.7 (7.3)	8.0 (4.3)	10.4 (6.3)	14.5 (7.4)	8.8 (5.3)	11.7 (7.0)	10.0 (8.5)	5.8 (3.6)	7.9 (6.8)
Sony CRE-C10 (n = 13)	7.8 (4.6)	6.6 (4.5)	7.2 (4.5)	8.9 (5.2)	7.8 (5.1)	8.3 (5.1)	6.5 (3.7)	5.4 (3.0)	5.9 (3.3)

Note: Measurements were performed at 250, 500, 1000, 2000, 3000, 4000, and 6000 Hz. dB = decibels; SPL = sound pressure level; SD = standard deviation.

**Table 2:** Experiment 2: Longitudinal evaluation of average differences between NAL-NL2 targets and real-ear measurements in dB SPL after initial fit and at follow-up intervals.

	Average Speech at 65 dB SPL Mean (SD)			Soft Speech at 55 dB SPL Mean (SD)			Loud Speech at 75 dB SPL Mean (SD)		
Visit	Left Ear	Right Ear	Both Ears	Left Ear	Right Ear	Both Ears	Left Ear	Right Ear	Both Ears
Initial fit (n = 15)	6.0 (3.5)	5.3 (4.5)	5.7 (3.3)	7.8 (3.6)	7.7 (4.6)	7.7 (3.4)	3.6 (3.1)	3.6 (4.8)	3.5 (3.1)
1–2-month follow-up (n = 13)	4.1 (3.3)	5.3 (2.7)	4.7 (2.4)	6.2 (3.8)	7.7 (2.6)	7.0 (2.8)	1.8 (2.5)	3.1 (2.5)	2.5 (1.7)
3–4-month follow-up (n = 11)	5.8 (3.3)	4.7 (3.8)	5.3 (3.0)	7.8 (3.6)	7.2 (4.0)	7.5 (3.4)	3.1 (2.7)	2.4 (2.8)	2.7 (2.4)
5–6-month follow-up (n = 11)	6.3 (4.5)	3.9 (3.0)	5.1 (3.1)	8.1 (2.9)	6.4 (3.1)	7.3 (2.6)	2.9 (2.7)	1.3 (2.5)	2.1 (2.3)
12–15-month follow-up (n = 10)	5.3 (2.6)	5.2 (2.6)	5.3 (1.9)	7.7 (3.1)	7.5 (2.4)	7.6 (2.4)	2.4 (2.0)	3.0 (2.6)	2.7 (1.4)

Note: Measurements were performed at 250, 500, 1000, 2000, 3000, 4000, and 6000 Hz. dB = decibels; SPL = sound pressure level; SD = standard deviation.

## Data Availability

The datasets generated and/or analyzed for the current study are available from the corresponding author upon reasonable request.

## Supplementary Materials

The following supporting information can be downloaded at: https://www.mdpi.com/article/10.3390/audiolres15010017/s1. Figure S1: Experiment 1: (A) Distribution of conventional pure tone audiometric frequencies for the left ear, (B) Distribution of conventional pure tone audiometric frequencies for the right ear, n = 43. Figure S2: Experiment 2: (A) Distribution of conventional pure tone audiometric frequencies for the left ear, (B) Distribution of conventional pure tone audiometric frequencies for the right ear, n = 15. Figure S3: Comparison of average prescribed NAL-NL2 targets and average real ear levels measured at 55 dB (soft speech) for ears combined across OTC hearing aids. Shading indicates the tolerance limits (±5 dB) from the mean for NAL-NL2 prescriptive targets. Figure S4: Comparison of average prescribed NAL-NL2 targets and average real ear levels measured at 75 dB (loud speech) for ears combined across OTC hearing aids. Shading indicates the tolerance limits (±5 dB) from the mean for NAL-NL2 prescriptive targets. Table S1: Experiment 1: Frequency and percentage of measurements within 5 dB from NAL-NL2 targets across ears for average speech (65 dB SPL). Table S2: Experiment 1: Frequency and percentage of measurements within 10 dB from NAL-NL2 targets across ears for average speech (65 dB SPL). Table S3: Experiment 1: Average Root Mean Square Error for 500-6000 Hz across six self-fitting over-the-counter heating aids. Table S4: Experiment 2: Average Root Mean Square Error for 500-6000 Hz after initial fit and at follow-up intervals.
