# Supplementary material for: Gain Analysis of Self-Fitting Over-the-Counter Hearing Aids: A Comparative and Longitudinal Analysis
Source: Audiol Res. 2025 Feb 13;15(1):17. doi: 10.3390/audiolres15010017 (PMC11851972; doi:10.3390/audiolres15010017)
Supplement: Supplementary file 1 [file audiolres-15-00017-s001.zip › audiolres-3356725-supplementary/audiolres-3356725-supplementary_V2/Supplementary Tables_Revised_Proofread.pdf]

Table S1. Experiment 1: Frequency and percentage of measurements within 5 dB from NAL-NL2 targets across ears for average speech (65 dB SPL)

Hearing aid	Frequency						
	250 Hz	500 Hz	1000 Hz	2000 Hz	3000 Hz	4000 Hz	6000 Hz
HP (n = 28 ears)	25 (89.3%)	18 (64.3%)	18 (64.3%)	9 (32.1%)	8 (28.6%)	8 (28.6%)	7 (25%)
Jabra (n = 30 ears)	24 (80%)	16 (53.3%)	16 (53.3%)	14 (46.7%)	8 (26.7%)	2 (6.7%)	7 (23.3%)
Lexie B2 (n = 30 ears)	22 (73.3%)	14 (46.7%)	11 (36.7%)	12 (40%)	10 (33.3%)	5 (16.7%)	12 (40%)
Lexie Lumen (n = 28 ears)	20 (71.4%)	18 (64.3%)	17 (60.7%)	15 (53.6%)	14 (50%)	6 (21.4%)	3 (10.7%)
Soundwave Sontro (n = 28 ears)	18 (64.3%)	11 (39.3%)	7 (25%)	8 (28.6%)	10 (35.7%)	3 (10.7%)	4 (14.3%)
Sony (n = 26 ears)	20 (76.9%)	12 (46.2%)	10 (38.5%)	11 (42.3%)	10 (38.5%)	5 (19.2%)	8 (30.8%)
All (n = 170 ears)	129 (75.9%)	89 (52.4%)	79 (46.5%)	69 (40.6%)	60 (35.3%)	29 (17.1%)	41 (24.1%)

Table S2. Experiment 1: Frequency and percentage of measurements within 10 dB from NAL-NL2 targets across ears for average speech (65 dB SPL)

Hearing aid	Frequency						
	250 Hz	500 Hz	1000 Hz	2000 Hz	3000 Hz	4000 Hz	6000 Hz
HP (n = 28 ears)	26 (92.9%)	26 (92.9%)	22 (78.6%)	14 (50%)	15 (53.6%)	16 (57.1%)	14 (50%)
Jabra (n = 30 ears)	27 (90%)	27 (90%)	24 (80%)	25 (83.3%)	20 (66.7%)	5 (16.7%)	11 (36.7%)
Lexie B2 (n = 30 ears)	27 (90%)	24 (80%)	16 (53.3%)	19 (63.3%)	19 (63.3%)	12 (40%)	18 (60%)
Lexie Lumen (n = 28 ears)	25 (89.3%)	25 (89.3%)	24 (85.7%)	22 (78.6%)	20 (71.4%)	13 (46.4%)	8 (28.6%)
Soundwave Sontro (n = 28 ears)	22 (78.6%)	22 (78.6%)	14 (50%)	19 (67.9%)	15 (53.6%)	6 (21.4%)	9 (32.1%)
Sony (n = 26 ears)	24 (92.3%)	21 (80.8%)	17 (65.4%)	23 (88.5%)	23 (88.5%)	13 (50%)	16 (61.5%)
All (n = 170 ears)	151 (88.8%)	145 (85.3%)	117 (68.8%)	122 (71.8%)	112 (65.9%)	65 (38.2%)	76 (44.7%)

Table S3. Experiment 1: Average Root Mean Square Error for 500-6000 Hz across six self-fitting over-the-counter hearing aids

	Average speech at 65 dB SPL Mean (SD)			Soft speech at 55 dB SPL Mean (SD)			Loud speech at 75 dB SPL Mean (SD)		
Hearing aid	Left ear	Right ear	Both ears	Left ear	Right ear	Both ears	Left ear	Right ear	Both ears
HP Hearing Pro (n = 14)	8.3 (5.0)	13.0 (7.7)	10.7 (6.8)	9.9 (6.8)	14.7 (8.8)	12.3 (8.1)	8.0 (7.4)	12.0 (7.5)	10.0 (7.6)
Jabra Enhance Plus (n = 15)	11.8 (4.8)	11.1 (3.9)	11.4 (4.3)	12.5 (5.6)	12.6 (3.8)	12.6 (4.7)	11.5 (4.6)	11.1 (3.4)	11.3 (4.0)
Lexie B2 (n = 15)	11.0 (5.5)	10.3 (5.1)	10.7 (5.2)	13.3 (6.1)	11.4 (5.4)	12.4 (5.8)	9.6 (4.5)	8.9 (4.6)	9.2 (4.5)
Lexie Lumen (n = 14)	11.2 (4.9)	9.0 (4.3)	10.1 (4.6)	10.6 (5.1)	9.0 (5.0)	9.8 (5.0)	11.8 (5.3)	9.6 (3.6)	10.7 (4.6)
Soundwave Sontro (n = 14)	15.2 (6.9)	11.3 (4.8)	13.2 (6.2)	16.6 (6.9)	11.4 (5.3)	14.0 (6.6)	13.5 (8.1)	9.9 (4.1)	11.7 (6.5)
Sony CRE-C10 (n = 13)	10.7 (4.8)	9.6 (4.3)	10.1 (4.5)	11.7 (4.9)	10.5 (4.5)	11.1 (4.7)	9.7 (4.0)	8.7 (2.9)	9.2 (3.5)

Note: Measurements were performed at 500, 1000, 2000, 3000, 4000 and 6000 Hz. dB = Decibels, SPL = Sound Pressure Level, SD = Standard deviation

Table S4. Experiment 2: Average Root Mean Square Error for 500-6000 Hz after initial fit and at follow-up intervals

	Average speech at 65 dB SPL Mean (SD)			Soft speech at 55 dB SPL Mean (SD)			Loud speech at 75 dB SPL Mean (SD)		
Visit	Left ear	Right ear	Both ears	Left ear	Right ear	Both ears	Left ear	Right ear	Both ears
Initial fit (n = 15)	9.2 (3.9)	8.9 (3.9)	9.1 (3.8)	11.2 (4.1)	10.8 (4.3)	11.0 (4.1)	7.6 (3.3)	8.1 (3.9)	7.8 (3.6)
1–2-month follow-up (n = 13)	7.3 (3.2)	8.2 (3.3)	7.8 (3.2)	9.5 (3.5)	10.6 (2.9)	10.1 (3.2)	6.0 (2.4)	6.5 (2.7)	6.3 (2.5)
3–4-month follow-up (n = 11)	8.7 (4.6)	7.6 (4.1)	8.2 (4.3)	10.7 (4.9)	9.8 (4.6)	10.2 (4.6)	6.8 (3.8)	6.1 (3.0)	6.5 (3.4)
5–6-month follow-up (n = 11)	9.0 (4.4)	7.0 (2.7)	8.0 (3.7)	11.0 (3.2)	9.1 (3.3)	10.1 (3.3)	6.9 (2.8)	5.5 (2.2)	6.2 (2.6)
12–15-month follow-up (n = 10)	8.2 (2.5)	8.6 (2.8)	8.4 (2.6)	10.5 (3.4)	10.6 (2.6)	10.5 (3.0)	6.1 (2.2)	7.2 (2.7)	6.7 (2.5)

Note: Measurements were performed at 500, 1000, 2000, 3000, 4000, and 6000 Hz. dB = Decibels, SPL = Sound Pressure Level, SD = Standard deviation
