# Supplementary figures and images for: Gain Analysis of Self-Fitting Over-the-Counter Hearing Aids: A Comparative and Longitudinal Analysis
Source: Audiol Res. 2025 Feb 13;15(1):17. doi: 10.3390/audiolres15010017 (PMC11851972; doi:10.3390/audiolres15010017)

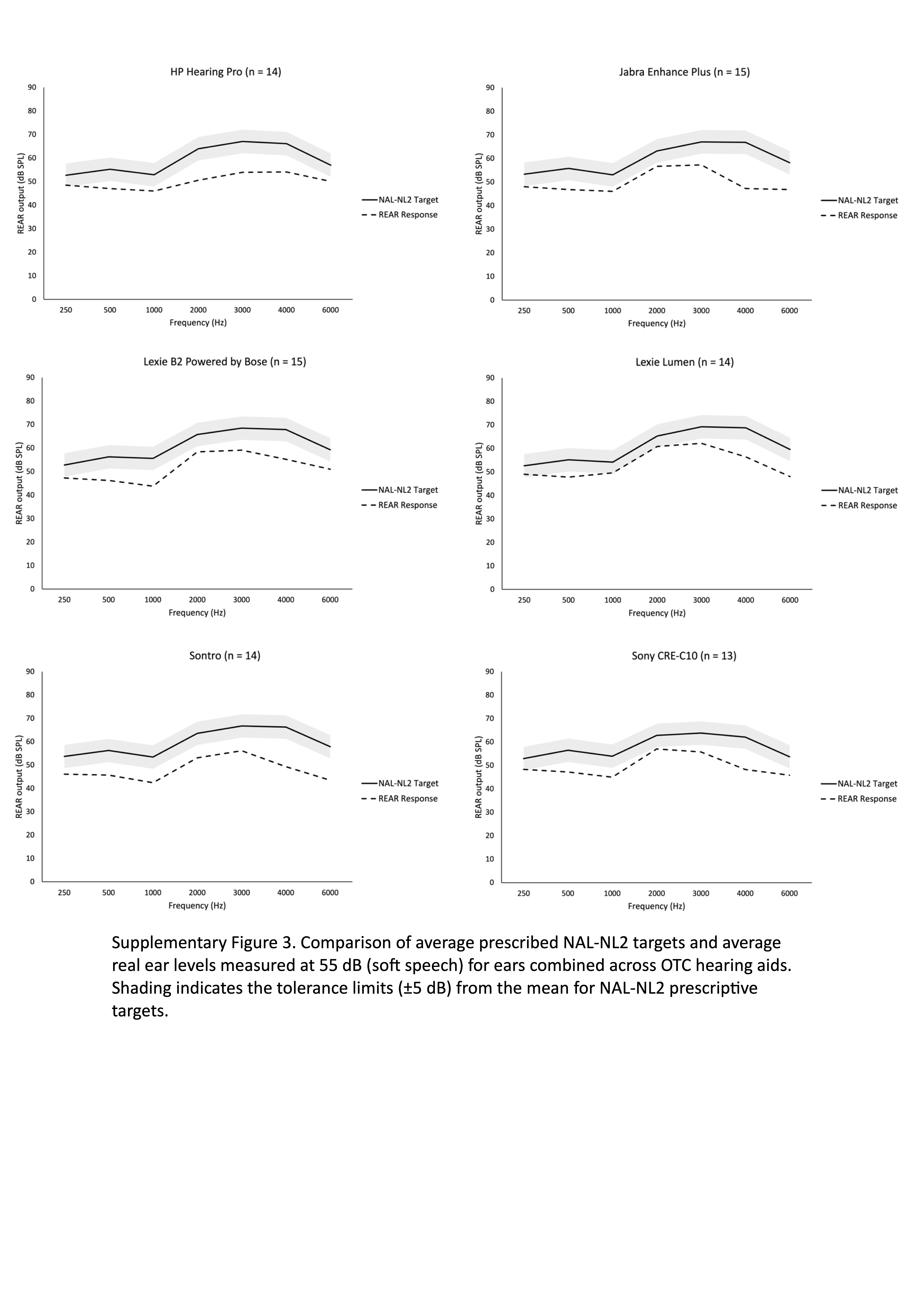

Supplement: Supplementary file 1 [file audiolres-15-00017-s001.zip › audiolres-3356725-supplementary/audiolres-3356725-supplementary_V2/Figure S3.tiff]

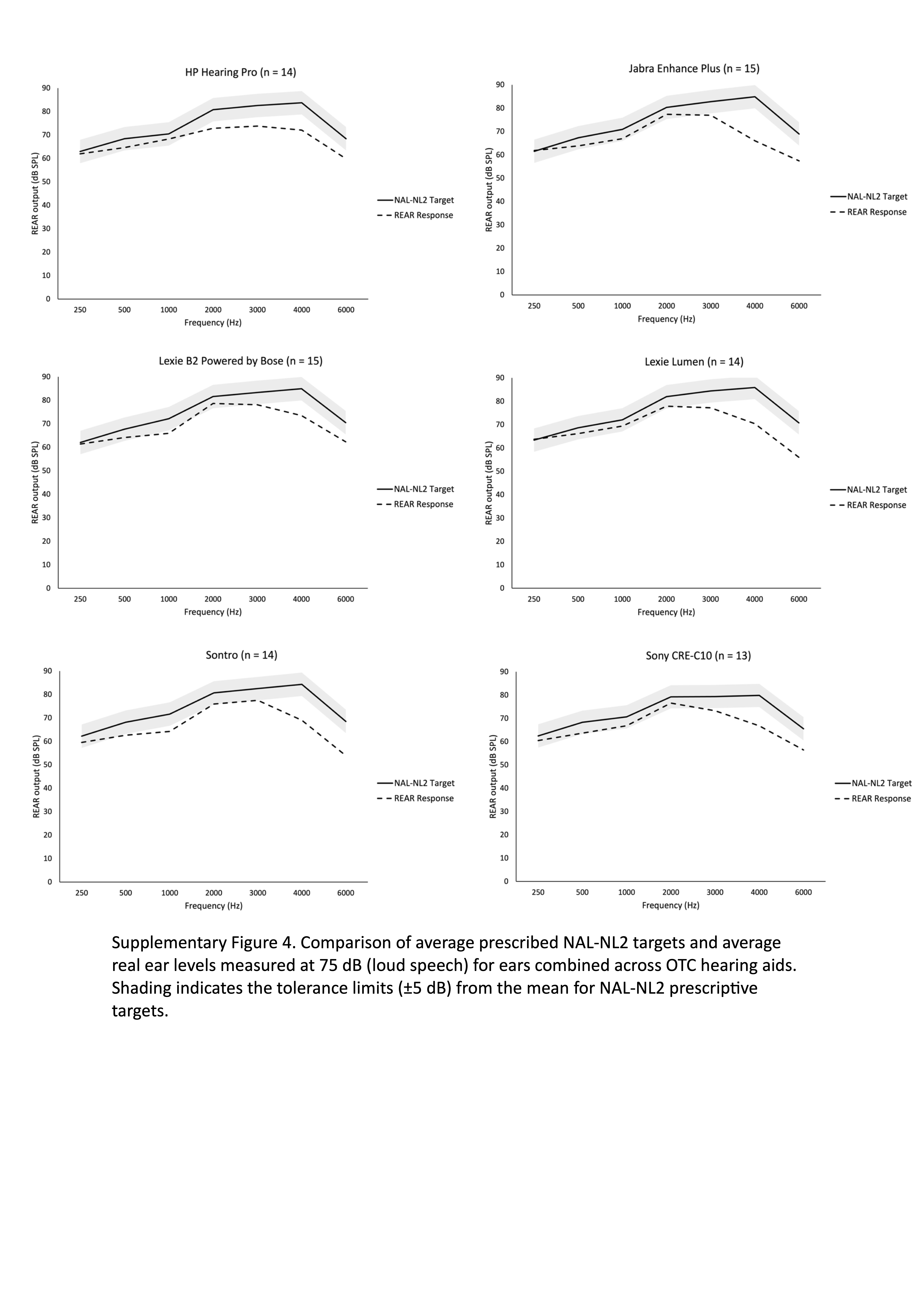

Supplement: Supplementary file 1 [file audiolres-15-00017-s001.zip › audiolres-3356725-supplementary/audiolres-3356725-supplementary_V2/Figure S4.tiff]
